# The sexual lives of people with disabilities within low- and middle-income countries: a scoping study of studies published in English

**DOI:** 10.1080/16549716.2017.1337342

**Published:** 2017-07-05

**Authors:** Mark T. Carew, Stine Hellum Braathen, Leslie Swartz, Xanthe Hunt, Poul Rohleder

**Affiliations:** ^a^ School of Psychology, University of East London, London, United Kingdom; ^b^ Leonard Cheshire Disability & Inclusive Development Centre, University College London, London, United Kingdom; ^c^ Department of Health, SINTEF Technology and Society, Trondheim, Norway; ^d^ Alan J. Flisher Centre for Public Mental Health, Department of Psychology, Stellenbosch University, Stellenbosch, South Africa

**Keywords:** Disability, sexuality, LMICs, sexual health, human rights

## Abstract

**Background:** Although approximately 80% of the global population of people with disabilities reside in low- and middle-income countries (LMICs), very little is known about their sexual lives due to a lack of empirical data. We aimed to provide a scoping review of English-language research conducted on disability and sexuality in LMICs.

**Objective:** Our research questions concerned *what* topics in disability and sexuality have (and have not) been investigated, *where* this research has been carried out, and *how* this research has been conducted.

**Methods:** A scoping review was conducted to examine the published English-language research literature on disability and sexuality within LMICs. We searched three electronic databases (PsycINFO, Web of Science, and PsycARTICLES) for research meeting these criteria published between 2000 and 2016 (inclusive). Through this search, we identified 103 articles.

**Results:** It is concluded that: (a) disability and sexuality research in African countries has focused predominantly on sexual abuse and violence or HIV, (b) the sexuality of people with disabilities within many LMICs has received little or no empirical investigation, and (c) there have been very few experimental studies on disability and sexuality conducted in LMICs in general.

**Conclusions:** Much remains unknown about the sexual health and sexual lives of the majority of people with disabilities, globally. Moreover, what has been done in certain contexts has tended to focus predominantly on vulnerabilities rather than emancipatory practices. Thus, urgent action is needed within LMICs on issues related to disability and sexuality to meet the goal of global optimal sexual health.

## Background

Sexual health, as currently understood, rests on the rights of individuals to freely express their sexuality in consensual relationships, to participate in activities such as marriage and starting a family, obtain detailed information about sexual issues, and access the highest possible standard of sexual healthcare [1]. Given that sexuality is viewed in human rights documents as a central aspect of being human [1], the realisation of sexual health, in common with other aspects of physical and mental wellbeing, should ideally be attainable for all persons. It is, therefore, unfortunate that this realisation remains far from ubiquitous worldwide, especially for people with disabilities, who often comprise the most marginalised and vulnerable group socially, sexually, and in relation to systems of care, including healthcare [2–4].

**Figure 1.**
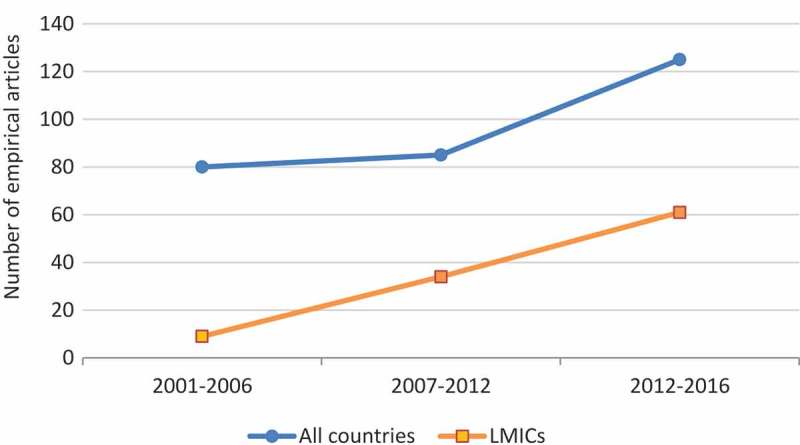
Empirical research articles published on disability and sexuality in the twenty-first century engaging with a rights perspective.

A review of the social and empirical evidence on disability and (a)sexuality, conducted in 2001, highlighted that people with disabilities tend to face disproportionate levels of difficulty in leading fulfilling sexual lives compared to people without disabilities, despite possessing the same sexual needs and desires [5]. The review identified that people with disabilities tend to encounter several barriers when expressing their sexuality and accessing sexual and reproductive healthcare, located at the individual (e.g. poor body image) [6], societal (e.g. negative attitudes) [7], and structural (e.g. inaccessible environments) [8] levels. In a context of increasing concern about disability rights globally [9], the field of disability and sexuality has continued to attract increasing interest from researchers in the twenty-first century. As of January 2017, a literature search within a leading scientific research database (Web of Science), using ‘disability’ and ‘sexuality’ and their synonyms, attests to the growing literature that has engaged with the area from a rights perspective (see Figure 1; These data are based on our own search of the literature, detailed in the ‘Methods’ section. To create the top line in Figure 1, we also ran another search while retaining literature published in high-income countries that also met the other inclusion criteria) and demonstrates the sizeable amount of empirical work that has been conducted since the 2001 review [5]. Whilst this work has generally focused on understanding or removing barriers to fulfilling sexual lives among people with disabilities in high-income countries, some studies have highlighted sites of concern of particular relevance to people with disabilities within low- and middle-income countries (LMICs) [10] (We classify countries as ‘low-’ and ‘middle-income’ based on the terminology of the World Health Organization and the World Bank [10]. Note that these organisations further divide middle-income countries into lower-middle and upper-middle. Our use of middle-income therefore encompasses both categories). For example, there is now much evidence to suggest that people with disabilities often experience additional barriers when accessing HIV treatment or preventative care, which may increase their vulnerability to the disease in contexts where HIV/AIDS is widespread, such as sub-Saharan Africa [11]. Other studies have highlighted the unique barriers that displaced people with disabilities face when trying to access sexual and reproductive healthcare within refugee camps [12].

Although comprehensive reviews of certain issues pertinent to the sexual lives of people with disabilities within LMICs have been conducted (e.g. disability and HIV) [13], discussion of the literature from these settings has largely been absent from previous general reviews of disability and sexuality [5,14]. This may in part be because the number of studies conducted in high-income settings continues to dwarf those conducted in low- and middle-income settings (see Figure 1).

In this article, we aimed to provide a scoping review concerning *what* topics in disability and sexuality have been investigated in LMICs, *where* this research has been carried out, and *how* this research has been conducted.

Specifically, our research questions are:

- What topics in disability and sexuality have been investigated in LMICs?
- Where has disability and sexuality research in LMICs been carried out?
- How has disability and sexuality research in LMICs been conducted?
- What are the gaps in the disability and sexuality literature in LMICs?

## Methods

### Search strategy and selection criteria

The goal of a literature review is to collect, analyse, and present available research evidence in a given field of interest. There are several methodologies for literature reviews, some more systematic and organised than others [15]. Both systematic reviews and scoping reviews use strict, transparent methods to identify, organise, and analyse all relevant literature in connection to a research question. However, while scoping reviews are well suited to give an overview of a potentially large field of research and to identify gaps in knowledge, systematic reviews are concerned only with the best-quality research within a narrow, clearly defined field of research and in relation to research questions of such nature. The objectives or research questions for a literature review determine the choice of method for the review [15]. For the purpose of the literature review presented in this article, a scoping review was the most suitable method, with its broad research questions and inclusion of a wide range of empirical evidence. We searched for English-language peer-reviewed journal articles containing empirical research on disability and sexuality that had been conducted within LMICs and published in 2000–2016 (inclusive). Specifically, we used a combination of search terms pertaining to *disability* (e.g. disab*, handicap*, bifida, crip*, sclerosis) and *sexuality* (e.g. sexual*, asexual*, romance, intim*, dating) within several scientific databases (PsycINFO, Web of Science, and PsycARTICLES). In addition to the database searches, we undertook a manual search of literature published in *African Journal of Disability, Disability & Rehabilitation*, and *Sexuality and Disability*.

We made the decision to constrain our search to literature published in 2000–2016 a priori because we wanted to provide a current overview of disability and sexuality in LMICs. Where possible, research conducted in high-income countries, research that was non-peer reviewed, or not in the English language was excluded using database options immediately after the search of each database was performed. Where this was not possible, we excluded cases that met these criteria, as well as cases that did not contain empirical research (e.g. commentaries), through inspection of the obtained abstracts. Moreover, given that the current conceptualisation of disability and sexuality is as a human rights issue [1], we excluded research that investigated the sexuality of people with disabilities from a predominantly medical/functional (e.g. sexual dysfunction in multiple sclerosis patients) or criminological/forensic (e.g. people with intellectual disabilities and sexual offending) perspective. We identified the majority of these cases through reading the obtained abstracts for key phrases (e.g. sexual dysfunction/functioning, sexual offenders/offending, sexual behaviour), though in some instances where there was a lack of clarity, the full paper was screened before the decision to exclude was made.

The provisional search of PsycINFO generated 1453 hits published in 2000–2016, which fell to 1283 when using database options to exclude non-peer-reviewed or non-English-language cases. The provisional search of Web of Science generated 1117 hits published in 2000–2016, falling to 89 when using database options to exclude cases that were conducted in high-income countries or were not peer-reviewed or in the English language. A provisional search of PsycARTICLES generated a far smaller amount of hits (< 100). Through this search, we identified 103 articles deemed appropriate to include in the scoping study. Sixty-one of these had been published between 2012 and 2016 (see Figure 1, bottom line).

### Data charting and collation

We developed a framework to extract relevant data from the obtained articles, including main topic(s), key participant group(s), sample *N*, and primary methodology (see the supplemental material). Themes were generated for each article based on an analysis of the article content by two of the authors. In subsequent discussion with the other authors, it was decided to group similar themes (e.g. self-attitude, knowledge, experiences) into topics, to best present an overview to the reader, in line with the objectives of a scoping review [15]. Sub-themes/topics (e.g. the participant group that attitudes belonged to) are also shown in the supplementary material to meet this aim.

### Data analyses and reporting

In addition to the tabulated supplementary material, we provide a general descriptive summary of the topics in what follows. Subsequently, addressing our first research question, we provide a narrative summary of key findings in each topic. The remaining research questions are addressed through descriptive analyses of the tabulated data and our consequent interpretative narrative, each presented in what follows.

### Role of funding source

The funder had no role in the study design, data collection, analysis, interpretation, writing, or submission of the manuscript.

## Results

### General description of topics

The empirical research identified through the scoping review spanned six overlapping topics (listed next). Within the identified articles, the most common topic was self-attitudes, knowledge, and experiences, found in 51 (49%) of the articles. An approximately equal amount of articles focused on community, parental, and professional attitudes, access to sexual and reproductive healthcare, or access to sexual education (range: *n* = 19–23; 18–22%). A minority of articles focused on sexual abuse/violence (*n* = 13, 12%) or intersectionality (*n* = 11, 10%). Twenty articles (19%) focused substantively on at least two topics.

### What topics have been researched in LMICs?

The key findings for each topic can be summarised as follows:

1. *Self-attitudes, knowledge, and experiences*. People with disabilities may engage in risky sexual behaviours and possess low levels of knowledge about safe sexual practices. Although many face challenges to leading sexual lives, such as low sexual self-esteem, people with disabilities are sexual beings and have the same needs as people without disabilities.
2. *Community, parental, and professional attitudes*. While the attitudes of people with disabilities are not uniformly negative, people with disabilities are not seen as fully sexual by community members. Parents of children with disabilities may be reluctant to accept the sexual desires of their offspring, especially if they have intellectual disabilities. Professionals may not know how best to address or teach common sexual topics when working with people with disabilities.
3. *Access to sexual and reproductive healthcare*. There are many barriers to accessing sexual and reproductive healthcare among people with disabilities, several of which are exacerbated by poor infrastructure and greater levels of poverty in LMICs. Examples include inaccessible healthcare facilities, poor provision of adequate transportation, and negative attitudes of healthcare providers.
4. *Access to sexual education*. There are also several barriers to accessing sexual education, for example, lack of provision of accessible information and lack of knowledge among teachers and parents.
5. *Sexual abuse/violence*. People with disabilities, particularly women, children, or individuals with intellectual disabilities, may be vulnerable to sexual abuse, exploitation, and violence.
6. *Intersectionality*. Disability and sexuality may intersect with other factors, such as gender and culture, to disadvantage certain people with disabilities in terms of their access to sexual healthcare or ability to live a fulfilling sexual life.

Additionally, these themes are frequently explored in the context of HIV and HIV prevention.

From these data, we noted that research conducted in certain regions tended to focus on certain issues. Specifically, we identified that, of the 63 studies conducted in Africa, 38 (60%) focused on disability and sexuality primarily in terms of sexual abuse and violence or HIV. Conversely, of the 40 studies conducted in other low- and middle-income settings, the range of issues investigated was much broader, with just 2 (5%) papers focused on sexual abuse and violence or HIV.

Addressing contexts of vulnerability that people with disabilities may experience in relation to their sexuality is of the utmost importance in promoting sexual health among this large, but marginalised, population, especially in LMICs where threats may be grave (e.g. HIV). To focus solely on the vulnerability of people with disabilities in the African region, however, is to skew knowledge about disability and sexuality in this context. In fact, there are many ways in which people with disabilities are not vulnerable and are able to live fulfilling sexual lives in Africa, as well as elsewhere in the world. For example, in South Africa, Chappell [16] found that youth with disabilities, who are not taught about sexuality by their parents, may develop hidden languages of sexual communication with their peers as a means of secretly resisting dominant cultural conceptions of their sexuality (and the assumption that they are asexual). Chappell et al. [17] also draw attention to the emancipatory ability of participatory research with people with disabilities (e.g. positioning them as co-researchers) to challenge problematic sexual constructions and to encourage the exercise of agency regarding sexual identities. Given that there is far less research conducted in low- and middle-income settings in general compared to high-income settings, there is certainly room for this emancipatory dialogue to emerge within the literature on disability and sexuality covering the African region.

### Where has research in LMICs been carried out?

Of the 103 articles identified in the scoping study, 27 (26%) reported research that had been conducted in South Africa, while 15 (15%) reported research that had been carried out in Turkey. Thus, literature from just two countries accounts for almost half of the articles present in the data-set. These are both upper middle-income countries with high levels of inequality, but with a research infrastructure bigger than those in many poorer countries. In order to obtain a truly global and accurate picture of the sexual lives of people with disabilities in low- and middle-income settings, the breadth of research must be expanded in order to encompass nations that, to date, have received little empirical attention. For example, we identified only one study in the data-set that had been conducted in mainland China, the largest nation in the world, though this could in part be an artefact of our searching only for articles published in English. The study [18] found that in rural China, marriage and procreation between non-disabled men and women who have intellectual disabilities are both frequent and accepted (at least on the surface), in part due to a gender imbalance between women and men and the stigmatisation attached to men of low socioeconomic status. Similarly, the only study [19] identified in the data-set conducted exclusively in Nepal suggests that rural Nepalese non-disabled women hold relatively positive attitudes towards the sexuality of people with disabilities, with nearly three quarters believing that people with disabilities can marry and have children. These examples illustrate the importance of extending the scope of current research to areas that have as yet received scant empirical attention, as this may elucidate discourses that challenge or extend extant knowledge of the sexual lives of people with disabilities in low- and middle-income settings.

### How has research on disability and sexuality been conducted in LMICs?

Fifty-six (54%) articles identified through the scoping review utilise quantitative methods of inquiry, while 47 (46%) utilise qualitative methods. The range of methods used across this body of research is diverse (e.g. case studies, life stories, interviews, focus groups, cross-sectional surveys, experimental studies). Each of these methods has the potential to contribute to social change in different ways, with narrative approaches, for example, providing case material with which policy-makers and service providers can identify and which may inform the revision of practices. On the other hand, larger-scale studies may be more influential in the context of the global imperative for evidence-based policy reform.

In wealthier countries, a number of studies (e.g. Hilberink et al. [20]) have conducted experimental evaluations of interventions designed to remove barriers to sexual and reproductive health rights for persons with disabilities, largely through evaluating training courses for healthcare professionals. We did not find such randomised studies in LMICs, but there was some quasi-experimental work. For example, Hanass-Hancock and Alli [21] conducted a formative (descriptive) evaluation of a real-world HIV workshop intervention. Randomisation and rigorous pre–post assessment were not possible in this study.

More systematic field experiments would be difficult to implement in this area, but there may be useful lessons to learn from other field experiments conducted in LMICs. For example, in the area of prejudice reduction, Paluck [22,23] conducted a field test of the impact of radio programmes featuring reconciliation messages on community attitudes in two LMICs, Rwanda and the Democratic Republic of Congo. Field tests of naturally occurring mass-media programmes may represent a promising avenue by which to explore the question of how to change problematic attitudes towards the sexuality of people with disabilities. Field experiments, furthermore, would allow researchers to test the efficacy of interventions (e.g. HIV training workshops) against real-world conditions, while retaining the ability to make causal inferences.

Vignette-based approaches may offer a useful method, both for eliciting data on attitudes towards disability and sexuality and for piloting methods to change such attitudes. In order to understand factors affecting attitudes, Morales et al. [24,25] presented a series of vignettes to participants. These varied the demographic (e.g. gender, partner age) and situational (e.g. contraceptive use) factors of a sexual relationship involving two people with a learning disability. They explored the determinants of the perceived acceptability of such relationships among community, family, and professional samples in Mexico. We found no studies using vignette methods to explore the impact of interventions to change attitudes towards disability and sexuality.

In summary, the literature we accessed is largely descriptive. Though more descriptive studies are needed, there is an even larger gap in terms of experimental research, particularly with regard to interventions. We acknowledge, though, that the question of the external validity and scalability of intervention research in LMICs, especially where there are poor infrastructure, a degree of social instability, and rapid social change, is complex. The question of how research impacts on policy, and policy on practice, is even more so. These broad practical, methodological, and strategic questions would need to be considered alongside more proximal questions of the design and evaluation of interventions [26,27].

## Discussion

There has been a growing international recognition of the human rights of people with disabilities, marked by the adoption of the United Nations Convention on the Rights of Persons with Disabilities (UNCRPD) [28]. The achievement of optimal sexual health for any human being is dependent on the realisation of basic human rights, such as the right to non-discrimination, to privacy and confidentiality, to be free from violence and coercion, as well as the right to education, information, and access to health services [29]. The UNCRPD [28] includes the right to optimal sexual health. That we know very little about the sexual health and sexual lives of the majority of the world population of people with disabilities is an urgent concern.

As with many areas, there is a dominance of research resources in the ‘global north’, yet many of the global health issues and concerns are in the ‘global south’. We cannot simply rely on the export of knowledge and interventions about disability and sexual health to areas where we know very little about the contextual realities of people with disabilities living there. Furthermore, although the term ‘persons with disabilities’ suggests a discrete population group, there is obviously a great deal of heterogeneity within this group. We need to know more about the various structural, attitudinal, and social-cultural barriers to sexual health for people with disabilities across diverse contexts, and for a range of disabilities. As Groce and colleagues [30] point out in relation to HIV prevention, treatment, and care for people with disabilities, a two-pronged approach is needed, with disability being included in ‘mainstream’ research and programmes, as well as targeted, disability-specific research and programmes. Furthermore, as the scoping study also reveals, where there has been some attention to disability and sexual health, it has tended to focus predominantly on vulnerabilities, and we need to know much more about emancipatory practices. This work cannot be achieved without building partnerships across sectors: disability organisations, researchers, policy-makers, and practitioners.

## Supplementary Material

Supplemental Data

## Paper context

We conducted a search of peer-reviewed literature published in English on disability and sexuality and identified two reviews that draw largely on literature from high-income countries. To our knowledge, this is the first scoping review to examine the published research literature on disability and sexuality specifically related to low- and middle-income countries. The findings suggest that much remains unknown about the sexual health and sexual lives of the majority of people with disabilities globally.
