# Supplementary material for: The sexual lives of people with disabilities within low- and middle-income countries: a scoping study of studies published in English
Source: Glob Health Action. 2017 Jul 5;10(1):1337342. doi: 10.1080/16549716.2017.1337342 (PMC5533142; doi:10.1080/16549716.2017.1337342)
Supplement: Supplemental Data [file zgha_a_1337342_sm0817.docx]

**Table 1. Empirical research on disability and sexuality identified through the scoping review**

| **Year published** | **Author(s)** | **Main topic (sub-topic)** | **Country of origin (world bank income classification)** | **Key participant group (sub-group)** | ***N*** | **Primary methodology(s)** |
| --- | --- | --- | --- | --- | --- | --- |
| 2002 | Cheausuwantavee | Self-attitudes/knowledge/experiences; Attitudes to disability and sexuality (community) | Thailand (upper middle) | People with and without (physical) disabilities | 100 (25 with disabilities) | Cross-sectional survey |
| 2002 | Padencheri et al. | Intersectionality (disability, gender, culture) | India (lower middle) | Parents (of people with intellectual disabilities) | 46 (23 pairs of parents) | Cross-sectional survey |
| 2004 | Potgieter & Khan | Self-attitudes/knowledge/experiences | South Africa (upper middle) | People with (physical) disabilities (youth) | 7 | Interviews |
| 2004 | Rodarte & Munoz | Self-attitudes/knowledge/experiences | Mexico (upper middle) | People with disabilities | 609 | Cross-sectional survey |
| 2004 | Smith et al. | Access to sexual and reproductive healthcare | Zambia (lower middle) | People with disabilities (women) | 20 | Interviews |
| 2005 | Yousafzai et al. | Access to sexual and reproductive healthcare; Access to sexual education (HIV) | Rwanda (low), Uganda (low) | People with disabilities (youth) | 123 | Focus groups |
| 2006 | Ozgul et al. | Self-attitudes/knowledge/experiences | Turkey (upper middle) | People with (physical) disabilities | 101 | Cross-sectional survey |
| 2006 | Wazikili et al. | Self-attitudes/knowledge/experiences (HIV) | South Africa (upper middle) | People with disabilities (youth) | 10 | Focus groups, Interviews |
| 2007 | Addlakhu | Self-attitudes/knowledge/experiences, Intersectionality (disability, gender, culture) | India (lower middle) | People with disabilities (youth) | 4 | interviews |
| 2007 | Wijesinghe et al. | Self-attitudes/knowledge/experiences | Sri Lanka (lower middle) | People with (physical) disabilities | 413 | Cross-sectional survey |
| 2008 | Allen-Leigh et al. | Attitudes to disability and sexuality (parental attitudes) | Mexico (upper middle) | Parents (of people with intellectual disabilities) | “2 focus groups” | Focus groups |
| 2008 | Braathen & Kvam | Self-attitudes/knowledge/experiences, Intersectionality (disability, gender, culture) | Malawi (low) | People with disabilities (women) | 23 | Interviews |
| 2008 | Enwerij & Enwerij | Access to sexual and reproductive healthcare (HIV) | Nigeria (lower middle) | People with (physical) disabilities | 227 | Cross-sectional survey |
| 2008 | Kvam & Braathen | Sexual abuse/violence, Intersectionality (disability, gender, culture) | Malawi (low) | People with disabilities (women) | 23 | Interviews |
| 2008 | Mendes et al. | Self-attitudes/knowledge/experiences | Brazil (upper middle) | People with and without (physical) disabilities | 90 (40 with disabilities) | Cross-sectional survey |
| 2009 | Babu et al. | Self-attitudes/knowledge/experiences | India (lower middle) | People with (physical) disabilities | 56 | Focus groups, interviews |
| 2009 | Hanass-Hancock | Intersectionality (gender, HIV) | South Africa (upper middle) | People with disabilities | 25 | Interviews |
| 2009 | Isler et al. | Self-attitudes/knowledge/experiences | Turkey (upper middle) | People with (learning) disabilities (youth) | 60 | Cross-sectional survey |
| 2009 | Phasa et al. | Sexual abuse/violence | South Africa (upper middle) | Professionals (various professional community roles) | 20 | Focus groups, interviews |
| 2009 | Wazikili et al. | Self-attitudes/knowledge/experiences (HIV) | South Africa (upper middle) | People with (physical) disabilities (youth) | 16 | Interviews |
| 2010 | Akkus et al. | Self-attitudes/knowledge/experiences | Turkey (upper middle) | People with (physical) disabilities | 33 | Cross-sectional survey |
| 2010 | Ayhan et al. | Attitudes to disability and sexuality (professional) | Turkey (upper middle) | Professionals (nursing students) | 125 | Cross-sectional survey |
| 2010 | Dabirian et al. | Self-attitudes/knowledge/experiences | Iran (upper middle) | People with (physical) disabilities | 14 | Interviews |
| 2010 | de Andrade & Baloli | Self-attitudes/knowledge/experiences (HIV) | South Africa (upper middle) | People with (sensory) disabilities | 7 | Interviews |
| 2010 | Dourado et al. | Self-attitudes/knowledge/experiences; Attitudes to disability and sexuality (of spouses) | Brazil (upper middle) | People with (mental health) disabilities and their spouses | 36 | Cross-sectional survey |
| 2010 | Maart & Jelsma | Self-attitudes/knowledge/experiences (HIV) | South Africa (upper middle) | People with (physical) disabilities (youth) | 91 | Cross-sectional survey |
| 2010 | Morales et al. | Attitudes to disability and sexuality (community) | Mexico (upper middle) | People without disabilities | 200 | Experiment |
| 2010 | Rohleder | Access to sexual and reproductive healthcare; Access to sexual education (HIV) | South Africa (upper middle) | Professionals (disability organisations) | 110 | Cross-sectional survey; Interviews |
| 2010 | Rohleder et al. | Access to sexual and reproductive healthcare; Access to sexual education (HIV) | South Africa (upper middle) | Professionals (disability organisations) | 14 | Cross-sectional survey |
| 2010 | Touko et al. | Access to sexual and reproductive healthcare (HIV) | Cameroon (lower middle) | People with (sensory) disabilities | 101 | Cross-sectional survey |
| 2011 | Akinci | Attitudes to disability and sexuality (professional) | Turkey (upper middle) | Professionals (nursing students) | 141 | Cross-sectional survey |
| 2011 | Akinci et al. | Attitudes to disability and sexuality (professional) | Turkey (upper middle) | Professionals (nursing students) | 161 | Cross-sectional survey |
| 2011 | Arulogun et al. | Sexual abuse/violence | Nigeria (lower middle) | People with disabilities (women, youth) | 167 | Cross-sectional survey |
| 2011 | Eide et al. | Self-attitudes/knowledge/experiences, Access to sexual and reproductive healthcare (HIV) | South Africa (upper middle) | People with disabilities | 285 | Cross-sectional survey |
| 2011 | Gomez et al. | Self-attitudes/knowledge/experiences | Philippines (lower middle) | People with (sensory) disabilities | 10 | Interviews |
| 2011 | Julia & Othman | Self-attitudes/knowledge/experiences, Access to sexual and reproductive healthcare | Malaysia (upper middle) | People with disabilities (women) | 33 | Cross-sectional survey |
| 2011 | McKenzie & Swartz | Attitudes to disability and sexuality (community); Intersectionality (culture) | South Africa (upper middle) | People without disabilities | 31 | Q methodology |
| 2011 | Morales et al. | Attitudes to disability and sexuality (parental, professional) | Mexico (upper middle) | Parents, caregivers (of people with learning disabilities) | 270 (120 parents) | Experimental |
| 2011 | Sayem et al. | Sexual abuse/violence | Bangladesh (lower middle) | People with (physical) disabilities (children) | 30 | Interviews |
| 2011 | Shumba & Abosi | Sexual abuse/violence | Botswana (upper middle) | People with (sensory) disabilities (children) | 31 | Cross-sectional survey |
| 2011 | Trani et al. | Access to sexual and reproductive healthcare | Sierra Leone (low income) | People with and without disabilities (women) | 235 | Cross-sectional survey |
| 2011 | Yazici et al. | Attitudes to disability and sexuality (professional) | Turkey (upper middle) | Professionals (healthcare students) | 325 | Cross-sectional survey |
| 2012 | Bal et al. | Self-attitudes/knowledge/experiences | Turkey (upper middle) | People with disabilities | 11 | Interviews |
| 2012 | Cardoso et al. | Self-attitudes/knowledge/experiences | Brazil (upper middle) | People with (physical) disabilities | 208 | Cross-sectional survey |
| 2012 | Mall & Swartz | Access to sexual education (HIV); Access to sexual and reproductive healthcare; Attitudes to disability and sexuality (professional) | South Africa (upper middle) | Professionals (teachers) | 21 | Focus groups; Interviews |
| 2012 | Oladunni | Access to sexual education | Nigeria (lower middle) | People with (physical) disabilities (youth) | 150 | Cross-sectional survey |
| 2012 | Oladunni | Access to sexual education | Nigeria (lower middle) | People with (physical) disabilities (youth) | 140 | Cross-sectional survey |
| 2012 | Olowookere & Adewole | Self-attitudes/knowledge/experiences; Intersectionality (HIV) | Nigeria (lower middle) | People with disabilities | 99 | Cross-sectional survey |
| 2012 | Pan and Ye | Self-attitudes/knowledge/experiences; Attitudes to disability and sexuality (community) | China (upper middle) | People with (intellectual) disabilities (women) | 3 | Life stories |
| 2012 | Rohleder et al. | Access to sexual education (HIV) | South Africa (upper middle) | Professionals (special education schools) | 34 | Cross-sectional survey |
| 2012 | Rohleder et al. | Self-attitudes/knowledge/experiences (HIV); Intersectionality (disability, gender) | South Africa (upper middle) | People with disabilities | 285 | Cross-sectional survey |
| 2013 | Javier et al. | Self-attitudes/knowledge/experiences[ | Colombia (upper middle) | People with and without (physical) disabilities | 84 (42 with disabilities) | Cross-sectional survey |
| 2013 | Hanass-Hancock et al. | Access to sexual and reproductive healthcare (HIV) | Southern, Eastern Africa (various) | People with disabilities | national populations | Cross-sectional survey (secondary data) |
| 2013 | McKenzie | Self-attitudes/knowledge/experiences; Attitudes to disability and sexuality (parents) | South Africa (upper middle) | People with disabilities and parents (of disabled children) | 17 (8 parents) | Focus groups; Interviews |
| 2013 | Mprah (a) | Access to sexual and reproductive healthcare; Access to sexual education | Ghana (lower middle) | People with (sensory) disabilities | 192 | Cross-sectional survey |
| 2013 | Mprah (b) | Self-attitudes/knowledge/experiences; Access to sexual education | Ghana (lower middle) | People with (sensory) disabilities | 192 | Cross-sectional survey |
| 2013 | Wickenden et al. | Self-attitudes/knowledge/experiences; Intersectionality (disability, gender, culture, HIV) | Zambia (lower middle) | People with disabilities (women) | 12 | Interviews |
| 2013 | Nareadi | Sexual abuse/violence | South Africa (upper middle) | Professionals (various professional roles at special education schools) | 32 | Interviews |
| 2013 | Simkhada et al. | Attitudes to disability and sexuality (community) | Nepal (low income) | People without disabilities (women) | 412 | Cross-sectional survey |
| 2013 | Sorsa | Self-attitudes/knowledge/experiences (HIV) | Ethiopia (low income) | People with disabilities (children) | 173 | Cross-sectional survey |
| 2013 | Susuman et al. | Self-attitudes/knowledge/experiences (HIV) | South Africa (upper middle) | People with disabilities | 1742 | Cross-sectional survey (secondary data) |
| 2014 | Aderemi et al. | Access to sexual and reproductive healthcare (HIV) | Ethiopia (low income) | People with disabilities | 412 | Cross-sectional survey |
| 2014 | Ahumuza et al. | Access to sexual and reproductive healthcare | Uganda (low income) | People with disabilities | 40 | Interviews |
| 2014 | Akyuz et al. | Self-attitudes/knowledge/experiences | Turkey (upper middle) | People with disabilities (women) | 12 | Interviews |
| 2014 | Altuntung et al. | Self-attitudes/knowledge/experiences | Turkey (upper middle) | People with (physical) disabilities (married women) | 10 | Interviews |
| 2014 | Astbury & Walji | Sexual abuse/violence | Cambodia (lower middle) | People with and without disabilities (women) | 354 (177 with disabilities) | Cross-sectional survey |
| 2014 | Chappell | Self-attitudes/knowledge/experiences | South Africa (upper middle) | People with disabilities (youth) | 19 | Focus groups |
| 2014 | Chappell et al. | Self-attitudes/knowledge/experiences | South Africa (upper middle) | People with disabilities (youth; as co-researchers) | 3 | Participatory research |
| 2014 | Chirawu et al. | Access to sexual education; Attitudes to disability and sexuality (professional) | South Africa (upper middle) | Professionals (teachers) | 99 | Cross-sectional survey |
| 2014 | De Beaudrap | Access to sexual and reproductive healthcare; Access to sexual education (HIV) | Southern Africa (various) | People with disabilities | 13 studies | Meta-analysis |
| 2014 | Devries et al. | Sexual abuse/violence | Uganda (low income) | People with and without disabilities (children) | 3706 | Cross-sectional survey (secondary data) |
| 2014 | Gurol et al. | Attitudes to disability and sexuality (parental) | Turkey (upper middle) | Parents (of people with intellectual disabilities) | 9 | Interviews |
| 2014 | Hanass-Hancock | Access to sexual education; Attitudes to disability and sexuality (professional) | South Africa (upper middle) | Professionals (teachers) | 49 | Cross-sectional survey |
| 2014 | Kassah et al. | Sexual abuse/violence, Intersectionality (disability, gender, culture) | Ghana (lower middle) | People with disabilities (women) | 5 | Focus groups; Interviews |
| 2014 | Munymana et al. | Self-attitudes/knowledge/experiences (HIV) | Rwanda (low) | People with (physical) disabilities | 157 | Cross-sectional |
| 2014 | Oksel & Gunduzoglu | Self-attitudes/knowledge/experiences | Turkey (upper middle) | People with (physical) disabilities (women) | 20 | Interviews |
| 2014 | Phasa & Myaka | Sexual abuse/violence | South Africa (upper middle) | Professionals (various professional community roles) | 12 | Interviews |
| 2014 | Swartz & Mall | Access to sexual education; Attitudes to disability and sexuality (professional) | South Africa (upper middle) | Professionals (various professional roles at special education schools) | 27 | Interviews |
| 2014 | Umoren & Adejumo | Self-attitudes/knowledge/experiences (HIV) | Nigeria (lower middle) | People with and without disabilities (youth) | 362 | Cross-sectional survey |
| 2014 | Van Rooy & Mufane | Self-attitudes/knowledge/experiences (HIV) | Namibia (upper middle) | People with disabilities | 19 | Focus groups |
| 2015 | Chappell | Self-attitudes/knowledge/experiences | South Africa (upper middle) | People with disabilities (youth) | 19 | Focus groups |
| 2015 | de Reus et al. | Access to sexual education (HIV); Attitudes to disability and sexuality (professional) | South Africa (upper middle) | Professionals (teachers) | 47 | Focus groups |
| 2015 | Hanass-Hancock | Access to sexual education (HIV); Attitudes to disability and sexuality (professional, people with disabilities) | Africa (various) | People with disabilities; Professionals (health workers) | 60 | Formative evaluation |
| 2015 | Ikuomola et al. | Self-attitudes/knowledge/experiences | Nigeria (lower middle) | People with (physical) disabilities | 28 | Interviews |
| 2015 | Kok & Akyuz | Attitudes to disability and sexuality (parental) | Turkey (upper middle) | Parents (of people with intellectual disabilities) | 144 | Interviews |
| 2015 | Mavuso & Maharaj | Access to sexual and reproductive healthcare; Self-attitudes/knowledge/experiences | South Africa (upper middle) | People with disabilities | 16 | Interviews |
| 2015 | Olaleye et al. | Self-attitudes/knowledge/experiences; Access to sexual education | Nigeria (lower middle) | People with disabilities (youth) | 103 | Cross-sectional survey |
| 2015 | Owiredu et al. | Self-attitudes/knowledge/experiences | Ghana (lower middle) | People with (physical) disabilities | 235 | Cross-sectional survey |
| 2015 | Parsons et al. | Self-attitudes/knowledge/experiences (HIV) | Zambia (lower middle) | People with disabilities | 32 | Interviews |
| 2015 | Peta et al. | Self-attitudes/knowledge/experiences; Intersectionality (disability, gender, culture, sexuality) | Zimbabwe (low) | People with disabilities (women) | 1 | Case study |
| 2015 | Salehi et al. | Self-attitudes/knowledge/experiences | Iran (upper middle) | People with (sensory) disabilities | 138 | Cross-sectional survey |
| 2015 | Salehi et al. | Self-attitudes/knowledge/experiences | Iran (upper middle) | People with (physical) disabilities | 183 | Cross-sectional survey |
| 2015 | Tanabe et al. | Access to sexual and reproductive healthcare; Intersectionality (disability, gender, culture) | Kenya (lower middle), Nepal (low), Uganda (low) | People with disabilities (refugees) | 287 | Cross-sectional survey |
| 2015 | Yilmaz et al. | Self-attitudes/knowledge/experiences | Turkey (upper middle) | People with (physical) disabilities (women) | 18 | Interviews |
| 2016 | Altundag & Calbayram | Access to sexual education | Turkey (upper middle) | People with (intellectual) disabilities (women, youth) | 54 | Experimental |
| 2016 | Ang & Lee | Access to sexual education; Attitudes to disability and sexuality (professional) | Malaysia (upper middle) | Professionals (teachers) | 314 | Cross-sectional survey |
| 2016 | Bornman & Rathbone | Access to sexual education | South Africa (upper middle) | People with (intellectual) disabilities (women) | 10 | Focus groups; Social stories |
| 2016 | Chappell | Self-attitudes/knowledge/experiences; Intersectionality (disability, gender, culture) | South Africa (upper middle) | People with disabilities (youth) | 22 | Focus groups |
| 2016 | Ganle et al. | Access to sexual and reproductive healthcare | Ghana (lower middle) | People with disabilities (women) | 72 | Focus groups; Interviews |
| 2016 | Mboua & Touko | Access to sexual and reproductive healthcare (HIV) | Cameroon (lower middle) | People with (sensory) disabilities | 317 | Cross-sectional survey |
| 2016 | Nyokangi & Phasa | Sexual abuse/violence | South Africa (upper middle) | People with (intellectual) disabilities (youth) | 16 | Focus groups; Interviews |
| 2016 | Tarkang et al. | Self-attitudes/knowledge/experiences; Access to sexual and reproductive healthcare (HIV) | Cameroon (lower middle) | People with (physical) disabilities | 10 | Interviews |
| 2016 | Tugut et al. | Attitudes to disability and sexuality (professional) | Turkey (upper middle) | Professionals (nursing students) | 255 | Cross-sectional survey |
